# Definitive hypofractionated radiotherapy for early glottic carcinoma: experience of 55Gy in 20 fractions

**DOI:** 10.1186/s13014-015-0505-6

**Published:** 2015-09-23

**Authors:** Ekin Ermiş, Mark Teo, Karen E. Dyker, Chris Fosker, Mehmet Sen, Robin JD Prestwich

**Affiliations:** Department Of Clinical Oncology, St. James’s Institute of Oncology, Level 4, Bexley Wing, Beckett Street, Leeds, West Yorkshire LS9 7TF UK

## Abstract

**Introduction:**

A wide variety of fractionation schedules have been employed for the treatment of early glottic cancer. The aim is to report our 10-year experience of using hypofractionated radiotherapy with 55Gy in 20 fractions at 2.75Gy per fraction.

**Methods:**

Patients treated between 2004 and 2013 with definitive radiotherapy to a dose of 55Gy in 20 fractions over 4 weeks for T1/2 N0 squamous cell carcinoma of the glottis were retrospectively identified. Patients with prior therapeutic minor surgery (eg. laser stripping, cordotomy) were included. The probabilities of local control, ultimate local control (including salvage surgery), regional control, cause specific survival (CSS) and overall survival (OS) were calculated.

**Results:**

One hundred thirty-two patients were identified. Median age was 65 years (range 33–89). Median follow up was 72 months (range 7–124). 50 (38 %), 18 (14 %) and 64 (48 %) of patients had T1a, T1b and T2 disease respectively. Five year local control and ultimate local control rates were: overall - 85.6 % and 97.3 % respectively, T1a - 91.8 % and 100 %, T1b - 81.6 and 93.8 %, and T2 - 80.9 % and 95.8 %. Five year regional control, CSS and OS rates were 95.4 %, 95.7 % and 78.8 % respectively. There were no significant associations of covariates (e.g. T-stage, extent of laryngeal extension, histological grade) with local control on univariate analysis. Only increasing age and transglottic extension in T2 disease were significantly associated with overall survival (both *p* <0.01). Second primary cancers developed in 17 % of patients. 13 (9.8 %) of patients required enteral tube feeding support during radiotherapy; no patients required long term enteral nutrition. One patient required a tracheostomy due to a non-functioning larynx on long term follow up.

**Conclusions:**

Hypofractionated radiation therapy with a dose of 55Gy in 20 fractions for early stage glottic cancer provides high rates of local control with acceptable toxicity.

## Introduction

Larynx cancer remains a common head and neck cancer, with 2360 cases diagnosed within the UK in 2011 [1]. The vocal cords (glottis) are the most commonly involved subsite, representing approximately 75 % of larnynx carcinoma [2]. Glottic carcinoma commonly presents early, and unlike many other head and neck cancer subsites, paucity of lymphatic drainage in the glottis mucosa conveys a low risk of lymphatic dissemination [3]. The aim of treatment for early glottic carcinoma is generally cure with laryngeal preservation and adequate/good voice quality. Definitive radiotherapy and transoral laser resection are both widely employed, with the choice depending on tumour factors including involvement of one or both cords, anterior commissure involvement, physician choice and expertise and patient preference. Reviews of the outcomes of radiotherapy and laser resections suggests comparable local control, and survival with similarly low risks of major complications [3, 4]; local control may be inferior after laser excision in cases with anterior commisure involvement [3]. Voice quality following laser resection is related to the extent of the resection [3, 5]. In the modern era, open partial laryngectomies are usually reserved to salvage local recurrences which remain suitable for laryngeal preservation [6].

A wide range of radiotherapy dose-fractionation schedules have been employed for the treatment of early glottic carcinoma [7–12]. In 2006, the Royal College of Radiologists (RCR) Radiotherapy Dose Fractionation guidance [13] commented that both conventional fractionation (2Gy per fraction) and hypofractionated schedules (16–20 fractions) were effective, although ‘noting that short fractionation regimens remain the minority practice internationally, with a less robust evidence based than that for conventional treatment’. Recommended schedules included 64–70Gy in daily 2Gy fractions over 6.5–7 weeks, 54–55Gy in 20 daily fractions over 4 weeks, and 50–52.5Gy in 16 daily fractions over 3 weeks for small volume disease only [13]. In a similar era, regimens employing 2.25Gy per fraction (to a total dose of 63–65.25Gy have also been recommended [7, 8].

Hypofractionation to minimise potential for tumour repopulation during radiotherapy is particularly appealing for early larynx in view of small field sizes, potentially allowing larger doses per fraction without excessive late morbidity [9]. Audits of UK practice have revealed that the use of larger doses per fraction, such as 2.75Gy in schedules of 55Gy in 20 fractions, are employed to treat a substantial proportion of patients within the UK for early larynx cancer [14, 15]. However, as highlighted in the RCR guidance [13] there is only limited published data documenting the efficacy of the more hypofractionated schedules. Importantly, these is little data available to support the use of similar schedules for the treatment of T2 glottic carcinoma, with the two main series reporting the use of hypofractionated schedules with fraction sizes >2.5Gy included only T1 disease [11, 12]. Here we report our 10 year experience of treating T1/2 glottic carcinoma with 55Gy in 20 fractions over 4 weeks.

## Methods

This is a single centre retrospective study. Patients treated between 2004 and 2013 with definitive radiotherapy for glottis laryngeal cancer were retrospectively identified from radiotherapy databases and electronic patient notes. Inclusion criteria were: biopsy proven invasive squamous cell carcinoma of the glottis, T1 or T2 N0 disease, intended definitive radiotherapy with a prescribed dose of 55Gy in 20 fractions over 4 weeks; patients with prior therapeutic minor surgery (eg. laser stripping, cordotomy) were included.

### Radiotherapy technique

Patients were immobilised with a beam directional shell. Conventional X-ray simulation was in the initial part of the study period until 2006; this was subsequently replaced by virtual CT simulation. The majority of treatment was delivered using lateral opposed photon fields. Standard field borders in treatment protocols were similar to those previously described [16]: 1) superior: mid thyroid notch, 2) inferior: bottom of cricoid cartilage, 3) anterior: 1 cm anterior to skin of neck, 4) posterior: anterior to vertebral body. Elective neck radiotherapy was not used for any patient. When shoulder position would have obstructed the delivery of lateral opposed fields, an anterior oblique arrangement was used. The CT data was loaded into the Helax-TMS VG-1B® (Nucleotron, Colombia, USA) treatment planning system (prior to 2008) and onto Oncentra MasterPlan® (Nucleotron, Colombia, USA) after 2008. Bolus was used at clinicians’ discretion, and was generally considered for disease of the anterior cord/commissure and if the patient’s neck was thin. Treatment was planned with 6MV photons, and prescribed to the International Commission on Radiation Units and Measurements (ICRU) reference point in accordance with the ICRU 50 recommendations [17], to a total dose of 55Gy in 20 fractions, 2.75Gy per fraction, delivered 5 days per week over 4 weeks. Treatment delivery was with a 6MV linear accelerator with 1 cm multileaf collimators (Elekta, Sweden).

### Follow up

Patients were typically reviewed weekly during treatment and then 6–8 weekly for 2 years post-treatment, with further follow up at increasing intervals for at least 5 years.

### Biologically effective dose calculation

The tumour biologically effective dose (BED) was calculated using the standard linear quadratic equation [18]:

$$ \mathrm{BED}=\mathrm{D}\left(1+\left(\mathrm{d}/\upalpha /\upbeta \right)\right)-\left(\left(\mathrm{L}\mathrm{n}(2)/\upalpha \right)\left(\left(\mathrm{T}-{\mathrm{t}}_{\mathrm{k}}\right)/{\mathrm{t}}_{\mathrm{p}}\right)\right) $$

In which: D = total dose (Gy), d = dose per fraction (Gy), α/β = linear (α) and quadratic (β) components of the linear quadratic model (Gy); T = overall treatment time (days); t_k_ = onset of accelerated repopulation time (days); t_p_ = average doubling time during accelerated repopulation (days). The parameters used for calculation of tumour BEDs were derived from prior radiobiological studies [18–20]: α/β = 10Gy; α = 0.3Gy^−1^; t_k_ = 22 days; t_p_ = 3 days. For late effects the standard linear quadratic equation was used [18, 21]:

$$ \mathrm{BED}=\mathrm{D}\left(1+\left(\mathrm{d}/\upalpha /\upbeta \right)\right) $$

In which for late responding tissues α/β = 3Gy [21].

### Statistical analysis

Statistical analysis was performed using SPSS version 16 (IBM, USA), STATA version 10 (Statacorp, USA), and Prism version 6 (Graphpad, USA). Survival and recurrence outcomes were calculated from the last date of of radiotherapy treatment. The following endpoints were used for assessment: local control, ultimate local control (including salvage treatment) regional control, cause specific survival (CSS) overall survival (OS), and were analysed using Kaplan–Meier product limit curves. Univariate Cox proportional hazards regression analysis was performed for the following factors: age, gender, smoking, alcohol consumption, histological differentiation, T stage (T1a versus T1b versus T2), anterior commissure involvement, supra-or sub-or trans-glottic extension, cord mobility, and prior laser therapy.

## Results

### Patient and treatment characteristics

One hundred thirty-two patients were identified who fulfilled the inclusion criteria for inclusion in this retrospective study. Patient, tumour and treatment characteristics are summarised in Table 1. The median follow-up time was 72 (range 7 to 124) months. 68 (52 %) and 64 (48 %) of patients had T1 and T2 disease respectively; all patients had N0 disease. All patients had biopsy proven squamous cell carcinoma. 112/132 (85 %) had undergone a contrast-enhanced staging CT of larynx, 2/132 (1.5 %) had undergone an MRI larynx, and 18/132 (14 %) did not undergo cross sectional imaging of the larynx (comprising 16 patients with T1a, one patient with T1b and one patient with T2 disease). 14 patients had undergone prior laser treatment for early glottic carcinoma; radiotherapy was delivered to 4 of these patients due to clinical evidence of residual disease in situ following laser treatment, and to 10 patients following biopsy proven recurrence after prior laser therapy. All patients received the intended prescribed radiotherapy dose of 55Gy in 20 fractions in 2.75Gy per fraction. The calculated tumour BED of the schedule is 67Gy. Median duration of radiotherapy was 28 days (range 25 to 35 days). Treatment duration was greater than 28 days in 2 patients (32 and 35 days); reason for delay was not documented for one patient and due to patient compliance for the other.

**Table 1:** Patient and disease characteristics (*n* = 132)

Characteristics		N of patients (range or %)
Mean Age		65 (33–89)
Sex	Male	117 (89 %)
	Female	15 (11 %)
Smoking	Never	17 (13 %)
	Current or former	106 (80 %)
	Unknown	9 (7 %)
Alcohol Use (moderate or heavy)	None	16 (12 %)
	Current or former	85 (64 %)
	Unknown	31 (23 %)
Performance status	0–1	116 (88 %)
	2	4 (3 %)
	3	2 (2 %)
	Unknown	11 (8 %)
T Stage	T1a	50 (38 %)
	T1b	18 (14 %)
	T2	64 (48 %)
Histology	Squamous cell	132 (100 %)
	Verrucous	1
	Papillary cell	1
	Spindle	1
Extension	Subglottic	34 (26 %)
	Supraglottic	17 (13 %)
	Transglottic	5 (4 %)
Anterior commissure involvement	Involved	59 (45 %)
	Non involved	72 (55 %)
	Missing	1 (1 %)
Cord mobility	Mobile	21 (16 %)
	Impaired	3 (2 %)
Grade	Well	32 (24 %)
	Moderate	49 (37 %)
	Poor	15 (11 %)
	Unknown	36 (27 %)
Prior laser	Yes	14 (11 %)
	No	118 (89 %)

### Disease and survival outcomes

131/132 (99 %) of patients had a complete clinical response to treatment; the patient with persistent disease underwent a salvage total laryngectomy. Five year actuarial local control rates were as follows: overall 85.6 %, T1a 91.8 %, T1b 81.6 % and T2 80.9 %. Ultimate local control rates (including successful salvage treatment) were: overall 97.3 %, T1a 100 %, T1b 93.8 % and T2% 95.8 %. Overall 5 year neck (regional) control was 95.4 %.

A total of 20/132 (15) patients experienced disease recurrence after the initial complete clinical response; median time to recurrence was 23 months (5 to 73). The patterns of recurrence are summarised in Table 2. Fourteen patients experienced local recurrence only after a median of 22 months (range 5–72) post radiotherapy. Of isolated local recurrences, 13/14 (93 %) were at the laryngeal subsite of the original primary tumour. Six patients developed regional neck recurrence, three of which was in combination with recurrence at the primary subsite.

**Table 2:** Recurrence patterns and management

Outcomes	N (% of total)
Complete response to radiotherapy	131 (99)
Persistent disease	1 (1)
Local Recurrence only	14 (11)
At primary subsite	13 (10)
Near primary subsite	1 (1)
Regional recurrence only	3 (2)
Combined locoregional recurrence	3 (2)
Salvage surgery	18 (14)
Laser cordotomy	2 (2)
Total laryngectomy ± neck diss.	13 (10)
Radical neck dissection	1 (1)
Radical neck dissection + brachytherapy	2 (2)

Table 2 includes details of salvage treatments. A total of 18 patients underwent salvage surgery. 3 patients with recurrence did not undergo surgical salvage due to limited performance status, patient choice and death from intercurrent illness. 12 of the 18 patients (67 %) who underwent salvage surgery are disease free and alive; 3 of 12 have a functional larynx.

**Fig. 1:**
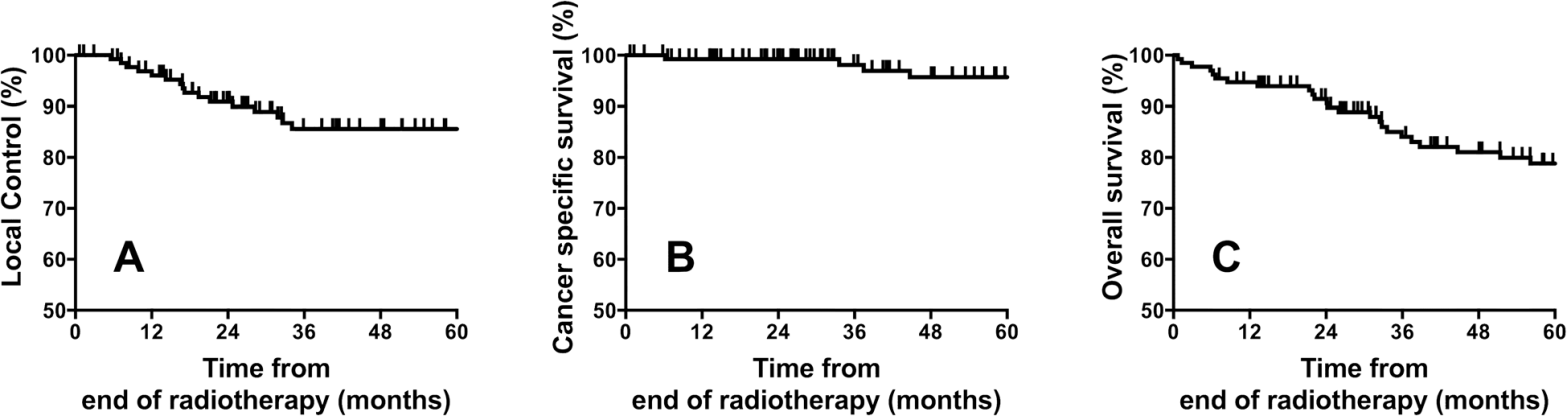
Kaplan Meier curves showing 5-year **a** local control, **b** cause specific survival and **c** overall survival

Five year CSS and OS were 95.7 % and 78.8 % respectively. A total of 36 (27.2 %) deaths occurred during follow up. The cause of deaths were: recurrent larynx cancer in 7 (5.3 %), second cancers in 14 (10.6 %) and death with other reasons in 15 (11.3 %) patients. Figure 1 illustrates 5-year local control, cause specific survival and overall survival outcomes.

Univariate analysis of covariates did not reveal any significant association with local control (all *p* >0.05). Only two covariates were significantly associated with overall survival: increasing age (*p* <0.01) and transglottic extension in T2 disease compared to T1 disease (Hazard ratio 4.43, 95 % confidence interval 1.47–13.39, *p* <0.01).

### Second malignancy

22 (17 %) patients developed second cancers during follow-up. Lung cancer was the most common site of a new primary tumour, occurring in 8 (36 %) patients. Details of second malignancies are listed in Table 3.

**Table 3:** Second malignancies in study group

Disease^a^	Incidence
Lung carcinoma	8 (6 %)
Gastric carcinoma	3 (2 %)
Colon carcinoma	2 (2 %)
Lymphoma	2 (2 %)
Lip, skin carcinoma	2 (2 %)
Hypopharynx	2 (2 %)
Soft tissue carcinosarcoma^b^	1 (1 %)
Renal cell carcinoma	1 (1 %)
Heamatologic malignancy	1 (1 %)
Adrenal tumor	1 (2 %)
Ovarian carcinoma	1 (1 %)

^a^Two patients had two malignancies

^b^In-field in right neck, 6 years post-radiotherapy

### Toxicity outcomes

Documented RTOG grade 3 skin toxicity occurred in 9 (6.8 %) of patients. RTOG grade 3 mucositis requiring enteral nutrition (via nasogastric tube feeding in all cases) occurred in 13 (9.8 %) patients. No deaths occurred within 90 days of completion of radiotherapy. Documented late toxicity in included post-cricoid stenosis in one patient managed with repeated dilatations, anterior glottic webbing in one patient, and one patient requiring a tracheostomy 3 years post radiotherapy with progressive laryngeal symptoms and no evidence of disease recurrence. No patients required long term enteral feeding.

## Discussion

Dose fractionation along with overall treatment time are expected to be key factors in the outcomes of definitive radiotherapy for early glottis cancer. Schedules with shortened overall treatment times have the potential advantage of minimising the impact of accelerated repopulation. Overall treatment time is known to be related to locoregional control for head and neck cancers; an analysis of two trials suggested that in node negative larynx cancer an additional dose of 0.8Gy/day is required to control tumour with increased treatment time [22]. An acceleration in treatment time can be achieved by either hypofractionation or hyperfractionation with multiple treatments per day. As previously described [21], radiobiological modelling based upon the linear quadratic model suggests similar log_10_ cell kill (as shown in Table 4) and a lower late-effects BED for a schedule of 55G in 20 fractions over 26 days compared with a conventionally fractionated schedule of 70Gy in 35 fractions over 46 days. For late effects (using an α/β of 3Gy for late responding tissues) the BED_3_ for a schedule of 55Gy in 20 fractions is 105.4Gy and for a schedule of 70Gy in 35 fractions is 116.6Gy respectively. This suggests a potential therapeutic gain for hypofractionation. Hypofractionation is a particularly appealing strategy for early glottic cancers with the limited size of the target volume and the consequently limited mucosal volume.

**Table 4:** Five year local control rates from studies employing hypofractionated radiotherapy for T1N0 and T20 squamous cell carcinoma of the glottis

Author/year	No. of patients	Dose (5 days/week daily fractionation unless stated)	Fraction size	OTT	tBED	Follow up/years	5 year LC T1a	5 year LC T1b	5 year LC T2
Current series	132 (*n* = 68 T1)	55Gy in 20	2.75	26	67.0	6	91.8 %	81.6 %	80.9 %
Moon et al. 2014 [26]	156 (*n* = 139 T1)	RCT:				5.6	5 years Local PFS		
		Conventional arm (*n* = 82):					T1/2:		
							77.8 % conv		
		66 in 33 for T1	2Gy	44	62.3		88.5 % hypo		
		70Gy in 35 for T2		47	64.7				
		Hypofractionated arm (*n* = 74):					T1 only:		
							80.3 % conv		
		63Gy in 28 (T1) 67.5Gy in 30 for T2	2.25Gy	38	64.9		90.1 % hypo		
				40	68.8				
							T1a only: 76.7 % conv		
							93 % hypo		
Kim et al. 2012 [27]	157 (n=125 T1 only)	70Gy in 35 (*n* = 64 pts)	2Gy	47	64.7	7.1	All T1		
							83 %		63 %
		67.5Gy in 30 (*n* = 93)	2.25Gy	40	68.8	3.8	95 %		61 %
Laskar et al. 2012 [28]	652 (T1 only)	50Gy in 15	3.3Gy	19	68.9	5	All T1: 90.6 % >3Gy per fraction 86.8Gy <3Gy per fraction (NS difference between fractionation schedules)		
		55Gy in 16	3.43Gy	22	73.9				
		60Gy in 24	2.5Gy	32	67.3				
		62.5 in 25	2.5Gy	33	69.6				
Jamshed et al. 2011 [30]	87 (*n* = 83 T1)	55Gy in 20	2.75	26	67.0	2.6	LRC 95 %	LRC 88 %	n/a
Chera et al. 2009 [16]	585 (*n* = 325)	63Gy in 28 (T1)	2.25Gy	38	64.9	12	94 %	93 %	T2a 80 %
		65.25Gy in 29 (T2)		39	66.8				T2b 70 %
		(39 % of series includes other schedules including hyperfractionation)							
Cheah et al. 2009 [11]	100 (T1 only)	50Gy in 16 fractions	3.125Gy	22	65.6	7	90 %	85 %	N/A
Short et al. 2006 [29]	145 (*n* = 102 T1)	60–66Gy in 30–33 (*n* = 51)	2	40–44	58.1–62.3	4.9	All T1 LRC 75 % conv		LRC 80 % (conv)
		52.5-55Gy in 20 (*n* = 94)	2.625–2.75	26	63.2–67.0		LRC 91 % hypo		LRC 81 % (hypo)
Yamazaki et al. 2006 [8]	180 (T1 only)	RCT: 60–66Gy in 30–33				Not stated	All T1		
		(66Gy if >2/3 of cord) (*n* = 89)	2Gy	40–44	58.1–62.3		LC 77 % conv		
		56.25Gy in 25 or 63Gy in 28 (>2/3cord) (*n* = 91)	2.25Gy	33–38	60.4–64.9		LC 92 % hypo		
Gowda 2003 [12]	200 (T1 only)	50–52.5Gy in 16	3.12–3.28Gy	22	65.6–68.7	5.8	93.1 %	89.1 %	

Conv = conventional fractionation

Hypo = hypofractionated

LC = local control

LRC = locoregional control

OTT = overall treatment time

PFS = progression free survival

tBED = tumour biologically effective dose

Our single institution series reports on outcomes using a schedule of 55Gy in 20 fractions with 2.75Gy per fraction with high rates of local control even after prior failure of laser therapy with acceptable toxicity. A high proportion of our series (48 % of patients) had T2 disease (which in the 2002 and 2007 TNM classifications [23, 24] includes the T2a and T2b stages of the 1998 classification [25]. Table 4 provides a comparison of 5-year local control rates from multiple studies [8, 11, 12, 16, 26–30] employing hypofractionated radiotherapy schedules for T1N0 and T2N0 squamous cell carcinoma of the glottis. Direct comparisons are limited by the heterogenous nature of the schedules used, variability in whether studies are exclusively of T1N0 disease or also include T2N0 disease and variable outcome measures. Similarly, Chera et al. previously documented 5-year local control rates from eleven large previous studies using a mix of conventional and hypofractionated schedules using the 1998 TNM classification which subdivides T2 disease into T2a and T2b based upon cord mobility. For T1a, T1b and T2a and T2b disease 5-year local control rates were reported in the order of 82–94 %, 80–93 %, 62–94 % and 23–73 % respectively; of note for T2a disease only a single study reported 5–year local control rates of >80 % [16]. By comparison, in our series 5-year local control rates were 91.8 %, 81.6 % and 80.9 % for T1a, T1b and T2 disease. These results appear particularly promising for T2 disease. Within the T2 group, five patients had supra-and subglottic extension and had an inferior outcome. Treatment failures were predominantly local with high rates of successful surgical salvage; lymph node recurrence was uncommon.

Hypofractionated regimens are widely used within the UK [13]. Despite this, few institutions have reported the use of accelerated hypofractionation with larger fraction sizes larger than 2.5Gy; in particular there is a paucity of data to support the use of these schedules for T2 disease. Gowda et al. [12] reported combined data from the Christie Hospital and Royal Marsden Hospital of 200 patients with T1 glottic cancer treated between 1989 and 1997 with 50–52.5Gy in 16 fractions over 3 weeks (fraction size 3.12–3.28Gy); overall 5–year local control was 93 %. Cheah et al. [11] reported the experience in Birmingham of a regimen of 50Gy in 16 fractions over 3 weeks in the treatment of 100 patients with T1N0 glottic carcinoma; overall 5-year locoregional control was 88 %. It should be noted that these series were restricted to T1N0 disease. Laskar et al. [28] also reported on acceptable local control of T1 glottic carcinoma with 50–55Gy in 15–16 fractions in a series comparing multiple fractionation schedules from a single institution. Short et al. [29] reported a retrospective comparison between 1986 and 1998 which included both T1/2 glottic carcinoma treated with 60–66Gy in 30–33 fractions compared with 52.5–55Gy in 20 fractions over 4 weeks in a later cohort of 94 patients (30 % T2 disease); for T1 disease, 5-year locoregional control was 75 % versus 95 % in favour of hypofractionation, although no difference was observed in for T2 disease (5-year locoregional control rates of 80 % versus 81 % respectively). A report examining the prognostic impact of pre-treatment haemoglobin concentration from Princess Margaret Hospital in Canada documented a 5-year relapse free rate of 81 % in 735 patients who received a standard dose of 50Gy in 20 fractions over 4 weeks, however in the subset with T2 disease 5-year relapse free rate was lower at 69 % [31].

Several randomised trials have examined the use of more modestly hypofractionationed schedules, using 2.25Gy per fraction. A Japanese trial by Yamazaki et al. randomised 180 T1 glottic cancer patients to 60–66Gy in 30–33 fractions (2Gy per fraction) versus 56.25–63Gy in 25–28 fractions (2.25Gy per fraction) (higher dose in both arms for tumours involving >2/3 of one cord); 5 year local control was 77 % versus 92 % (*p* = 0.004); the authors’ conclusion was that the higher dose per fraction of 2.25Gy with a shorter overall treatment time offered superior local control [8]. A randomised controlled trial from South Korea [26] compared a conventional 2Gy per fraction arm (66Gy in 33 fractions for T1 and 70Gy in 35 fractions for T2) with a hypofractionated 2.25Gy per fraction arm (63Gy in 28 fractions for T1 and 67.5Gy in 30 fractions for T2); the study closed prematurely due to poor accrual with 156 of a planned 282 patients; 5-year local progression free survival was 77.8 % versus 88.5 % (non-significant).

In addition, several retrospective series report on the use of 2.25Gy fraction sizes. A large retrospective series of 585 patients treated at the University of Florida for T1/2 glottic cancer was recently reported; 61 % of patients received ≥2.25Gy/fraction. As detailed in the paper, overall local control rates compared favourably with other reported series with 5 year local control rates of T1a 94 %, T1b 93 %, T2a 80 % and T2b 70 % (using the 1998 5th edition of the American Joint Committee on Cancer (AJCC) staging guidelines for early-stage SCCA of the glottis [25]), and on multivariate analysis overall treatment time >41 days was associated with inferior outcome. A series of 398 patients treated at the University of California was reported in 1997, including a range of fraction sizes; 5-year local control was 85 % for T1 and 70 % for T2. Fraction size of ≥2.25Gy was a significant factor in local control for T2 tumors [9]. Increased fraction size appeared beneficial independent of total dose and treatment time [9, 32]. Kim et al. [27] reported a retrospective analysis outcomes of a series of 157 patients with T1/2 glottic carcinomas treated with either 70Gy in 35 fractions or 67.5Gy in 30 fractions; disease free survival was significantly superior in the 2.25Gy/fraction arm. Overall this increasing body of evidence have established hypofractionation as the standard of care for definitive radiotherapy treatment of T1/2 glottic carcinomas, providing increased local control with acceptable toxicity.

In addition to the radiobiological perspective, the use of hypofractionation is appealing from both health economics and patient perspectives. Hypofractionated radiotherapy reduces both treatment time, number of fractions delivered and consequently cost; this eases the burden of treatment upon institutions. In addition, patients potentially benefit from the convenience of shorter schedules with fewer treatment visits required.

Hyperfractionation is an alternative approach to accelerate treatment schedules. The recently reported RTOG 9512 hyperfractionation study of 250 patients with T2N0 glottic carcinoma recruited between 1996 and 2003 compared a conventional arm of 70Gy in 35 daily fractions with 79.2Gy in 66 fractions of 1.2Gy bi-daily fractions; 5-year local control rate was 70 % versus 78 % (non-significant, *p* = 0.14) [10]. The 5-year local control rates in our series of 80.9 % is superior that achieved in the hyperfractionation arm of this study. The absence of a significant benefit of hyperfractionation, along with the practical challenges of implementing bi-daily treatment, suggests that hyperfractionation will not replace hypofractination as a standard treatment approach.

In our series, we found an 17 % of patients developed another primary cancer. This is similar to the rate of 21 % reported by Cheah et al. [11], 22 % by Khan et al. [33], 20 % and 22 % by Franchin et al. [34]. In the RTOG 9512 study, 20 % of deaths were due to a second primary [10]. Since smoking is the major aetiological factor for larynx carcinoma, these data serve to emphasise the importance of smoking cessation interventions and highlight this a potential group of patients for targeting screening interventions.

There are several limitations to our series. In common with other series, it is difficult to determine whether the ‘recurrence’ on long term follow up represents local failure or the development of a new primary; for the purposes of analysis we have classified these cases as recurrence Comparison with other series is complicated by the range of differing outcome measures reported, differing proportions of T1 versus T2 disease, and some previous series dividing T2 disease into T2a and T2b according to older versions of the TNM classification [16]. In addition to tumour control, voice quality is an important outcome in the treatment of larynx cancer, and in view of the retrospective nature of this analysis, no data is available. A previous study of 25 patients has previously shown voice outcomes improved over period 12 months compared with pre-treatment following treatment with a hypofractionated schedule (50Gy in 16 fractions) [35]. In common with other series, late toxicity is patchily documented. There is current interest in the potential for intensity modulated radiotherapy techniques to offer carotid artery sparing [36]; it was not possible to reliably document the long term risk of cerebrovascular events with conventional radiotherapy in this series.

## Conclusion

In summary, the optimum fraction size/dose and treatment time for early glottic cancer has yet to be established, although in the published literature hypofractionated accelerated radiotherapy schedules appear to be highly effective. This series demonstrates that a schedule of 55Gy in 20 fractions over 4 weeks offers high rates of local control with acceptable long term toxicity for both T1 and T2 disease.

## Abbreviations

CCS: Cause specific survival
OS: Overall survival
RCR: Royal college of radiologists
RTOG: Radiotherapy oncology group
